# The In Vitro Antitumour Activity of Novel, Mitochondrial-Interactive, Gold-Based Lipophilic Cations

**DOI:** 10.1155/2008/864653

**Published:** 2008-03-27

**Authors:** Sherika Mahepal, Richard Bowen, Messai Adenew Mamo, Marcus Layh, Constance Elizabeth Jansen van Rensburg

**Affiliations:** ^1^Department of Pharmacology, University of Pretoria, P.O. Box 2034, Pretoria 0001, South Africa; ^2^School of Chemistry, University of the Witwatersrand, Private Bag 3, Johannesburg 2050, South Africa

## Abstract

In this study we compared the effects of two previously described antimitochondrial gold complexes, that is, [A] [Au(dppe)_2_]Cl and [B] [Au(d4pype)_2_]Cl with two novel lipophilic cations, that is, [C] [Au(dpmaaH_2_)(dpmaaSnMe_2_)]Cl and [D] [Au(dpmaaSnMe_2_)_2_]Cl as antimitochondrial agents. The results of this study indicate that [C] and [D] have intermediate partition coefficients and exhibited a selective uptake by cells. They exhibited a higher selectivity for the various cell lines than [A] but were more cytotoxic than [B]. There is a significant correlation between the cytotoxic potential of [A], [B], [C], and [D] and their octanol/water partition coefficients in both MCF-7 (breast cancer) and MCF-12A (nonmalignant breast) cells, whereas their cytotoxic potential and ability to induce the release of cytochrome c correlated only in the case of the MCF-12A cells. Complexes [C] and [D] are promising new chemotherapeutic drugs. These compounds target the mitochondrial membranes of certain cancer cells exploiting the differences between the mitochondrial membrane potential of these cells and normal cells. Although the concentrations of these compounds necessary to eradicate cancer cells are very high, the results provide a basis for the synthesis of a new family of compounds with intermediate partition coefficients compared to [A] and [B] but with increased activity against cancer cells.

## 1. INTRODUCTION

**Figure 1:**
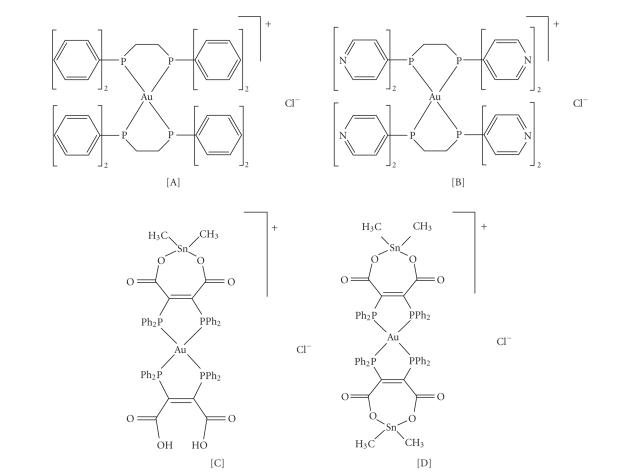
Structure of the experimental compounds: [Au(dppe)_2_]Cl [A], [Au(d4pype)_2_]Cl [B], [Au(dpmaaSnMe_2_)(dpmaaH_2_)]Cl [C], and [Au(dpmaaSnMe_2_)_2_]Cl [D]. (50 *μ*M).

The successful chemotherapy of cancer has been hindered by the acquired resistance of tumour cells to clinical drugs, which has necessitated the use of multidrug therapy with a higher potential for adverse drug effects. The use of relatively nonselective compounds results in the eradication of normal cells in addition to tumour cells. Previous studies have indicated that [Au(dppe)_2_]Cl (see Figure 1) exhibits antitumour activity in a wide range of tumour models in mice [1]. Since the lipophilic cationic properties of [Au(dppe)_2_]Cl promote its nonselective uptake into the mitochondria of all cells, strategies were adopted to synthesize more hydrophilic analogues that retained antitumour activity while being less toxic to the mitochondria.

The lipophilicity of gold(I) phosphine complexes can be controlled by an appropriate substitution at the quaternary centre or alkyl backbone that enables the modification of the compound to achieve greater selectivity for tumour cells [2]. This has led to the synthesis of the 3- and 4-pyridyl substituted analogues of [Au(dppe)_2_]Cl by the replacement of some or all of the phenyl substituents in dppe with hydrophilic pyridyl groups [3]. The lipophilicity of these tetrahedral bis(dipyridylphosphino) gold(I) complexes plays a key role in determining their cellular uptake [4]. It is therefore necessary to retain the lipophilicity of the compound within a certain octanol-water coefficient range in order to facilitate the uptake of a drug and determine the degree of protein binding [2].

Although the precise mechanism of action of these gold-containing lipophilic aromatic compounds is poorly understood, several studies have suggested a mitochondrial mode of action as well as the occurrence of DNA strand breaks and DNA-protein crosslinks in tumour cells [1, 2, 4, 5].

Tumour cells possess one of the highest mitochondrial transmembrane potentials [6, 7], which may be due to the inability of tumour cells to use mitochondria to fulfil their demands for ATP. Normal cells rely on oxygen consumption and oxidative phosphorylation for ATP production that takes place in the mitochondria, whereas tumour cells rely primarily on glycolysis for ATP production that takes place in the cytosol of the cell [4]. Lipophilic cations that possess a positive charge are able to penetrate the hydrophobic barriers of the plasma and mitochondrial membranes [8]. These compounds accumulate in the mitochondria in response to a negative transmembrane potential, which is the rationale behind the use of lipophilic cations as anticancer agents.

According to Davis et al. the uptake of a lipophilic cation is dependent on both the plasma and mitochondrial membrane potentials. The plasma membrane preconcentrates the drug in the cytoplasm and the mitochondrial membrane, in turn, concentrates the drug within the mitochondria [9]. These facts provide the perfect opportunity for drug accumulation in tumour cells and the feasibility for the use of tumour selective antimitochondrial compounds in human cancer chemotherapy.

The [Au(d4pype)_2_]Cl complex (see Figure 1), which is hydrophilic, exhibited a higher tumour uptake and a smaller in vivo liver/tumour ratio in treated mice with advanced Colon 38 adenocarcinoma tumours than [Au(dppe)_2_]Cl [10]. McKeage et al. [4] corroborated that compounds with an intermediate lipophilicity when compared to more lipophilic or hydrophilic compounds displayed significant antitumour activity, less dose-limiting toxicity, and a higher plasma concentration of gold. A correlation between the lipophilicity of the drug and the degree of selectivity and cytotoxic potency of the compounds has been established [10]. This denotes that an enhanced selectivity is achieved with greater hydrophilicity but an increase in the potency of the drug can be observed with a greater lipophilicity.

The common occurrence of drug-resistant tumour cells and the lack of selectivity of cancer drugs in differentiating between tumour cells and normal cells are two overriding problems in cancer chemotherapy [10]. In an attempt to combat this lack of selectivity, an investigation was initiated into two new aromatic cations, that is, [Au(dpmaaSnMe_2_)(dpmaaH_2_)]Cl [C] and [Au(dpmaaSnMe_2_)_2_]Cl [D] (see Figure 1). The simultaneous presence of gold and tin in aromatic cations should offer the advantage of reducing the development of resistance in cancer cells.

## 2. MATERIALS AND METHOD

### 2.1. Octanol/water partition coefficient

60 *μ*M stock solutions of [Au(dppe)_2_]Cl [A], Au(d4pype)_2_]Cl [B], [Au(dpmaaSnMe_2_)(dpmaaH_2_)]Cl [C], and [Au(dpmaaSnMe_2_)_2_]Cl [D] were prepared in octanol (Merck, Darmstadt, Germany). Water-saturated octanol and octanol-saturated water were prepared by shaking equal volumes of octanol and water for 15 minutes and allowing the mixture to separate into the respective phases for 20 hours. The absorbance of the initial drug concentration in the water-saturated octanol at 20 *μ*M, 40 *μ*M, and 60 *μ*M was analysed by UV spectrophotometry. Five millilitres of the octanol-saturated water were then added to 5 ml of the drug/octanol solutions to obtain a final volume of 10 ml. These solutions were shaken vigorously for 15 minutes, thereafter they were left to separate into an octanol and aqueous phase for 20 hours. The aqueous phase was separated ensuring that there was no contamination from the octanol phase, and each of these solutions was analysed by UV spectrophotometry in 1 cm quartz cuvettes to obtain the absorbance of the compounds.

### 2.2. Cell cultures

Cytotoxicity assays were performed on the following cell cultures: human ovarian carcinoma (A2780)(ECACC93112519) and its cisplatin-resistant subline (A2780cis)(ECACC93112517), breast carcinoma (MCF-7)(HTB-22), colon cancer (CoLo 320 DM)(CCL-220), cervical carcinoma cells (HeLa)(CCL-2), mouse melanoma (B16)(ECACC92101203), primary human fibroblasts (CCL-171), and a breast nontumourogenic cell line (MCF-12A)(CRL-10782). A2780, A2780cis and CoLo 320 DM were maintained in RPMI; B16, HeLa cells, and the primary fibroblasts were maintained in EMEM. B16 cells were maintained in DMEM. MCF-7 cells were maintained in DMEM supplemented with 2% nonessential amino acids and MCF-12A cells were maintained in a 1 : 1 mixture of DMEM and HAMS F12 medium with hydrocortisone (10 mg/mL), cholera toxin (1 mg/mL), insulin (20 mg/mL), 10% foetal calf serum (FCS), and epidermal growth factor (100 mg/mL). All media were supplemented with 1% solution of penicillin and streptomycinand 10% heat inactivated FCS.

### 2.3. Cell growth assay

A metabolic assay based on the reactivity of MTT (3-[4,5-dimethylthiazol-2-yl)-2,5-diphenyl-tetrazolium bromide), originally described by Mosmann [11] with modifications [12], was used to determine the effects of the experimental compounds on cell growth after a 7-day treatment period in 96 well-round bottom microtiter plates.

### 2.4. Cellular uptake of gold

MCF-7 and MCF-12A cells were prepared in supplemented DMEM at a stock concentration of 5 × 10^6^ cells/mL. The cells were exposed to the various compounds at concentrations that lead to 80–90% of cell death in the above-mentioned assay for 30 minutes at 37°C, centrifuged for 10 minutes at 185 g, and the pellets washed with Hank's balanced salt solution (HBSS). The gold content of the pellets was determined on an inductively coupled plasma mass spectrometer (ICP-MS) using standard procedures.

### 2.5. Release of cytochrome c

A suspension of MCF-7 and MCF-12A cells (2 × 10^6^ cells/mL) was prepared in supplemented DMEM. The cells were exposed to a concentration of the various compounds that lead to 80–90% of cell death for 30 minutes at 37°C, centrifuged for 10 minutes at 185 g, and the pellets washed with HBSS and assayed according to the manufacturers' instructions for cytochrome c using an assay kit (Sigma Chemical Co, St. Louis, Mo, USA).

### 2.6. Statistical analysis

The Pearson correlation coefficient (±95% confidence interval) was calculated and used to determine correlations between the various assay results.

## 3. RESULTS

### 3.1. Octanol/water partition coefficient

The average octanol/water partition coefficient values and the mean log octanol/water partition coefficient values are summarised in Table 1. The results indicate that [A] has a mean log octanol/water partition coefficient of 1.07, making it a lipophilic compound whereas [B] is hydrophilic with a very low log octanol/water partition coefficient of − 1.57. The octanol/water partition coefficients of both [C] and [D] (−0.012 and 0.09, resp.) are intermediate to that of [A] and [B].

### 3.2. Cytotoxicity assays

The IC_50_ values of the compounds for the various cell lines are summarised in Table 2. All eight cell cultures were more sensitive to [A] than any of the other compounds tested. [B] was more selective for certain cell types such as MCF-7. This cell line was 8-9 times more sensitive for this compound than the colon cancer cell line (CoLo 320DM) and the primary fibroblast culture. [C] and [D] also exhibited some selectivity for certain cell types viz [D] acted on the B16 cells at a concentration that was 9.5 times lower than what was necessary to inhibit the growth of CoLo 320DM.

### 3.3. Cellular uptake of experimental compounds

The average amount of gold taken up by MCF-7 and MCF-12A cells after treatment with the various experimental compounds is summarised in Table 3. The cells treated with [A] and [B] accumulated almost twice the percentage of gold than cells treated with [C] and [D].

### 3.4. Release of cytochrome c

The average percentage of cytochrome c release by MCF-7 and MCF-12A cells is summarised in Table 4. The results indicate that the outer membrane of mitochondria of cells treated with [A] incurred the greatest amount of damage as only 2-3% of the cells had undamaged mitochondrial membranes after treatment. There were no significant differences between the sensitivity of the mitochondrial membranes of MCF-7A and MCF-12A for the experimental drugs.

### 3.5. Pearson's correlation coefficient

(i) There is a significant correlation (*P* < .05) between the cytotoxic potential of [A], [B], [C], and [D] and their octanol/water partition coefficients in both MCF-7 and MCF-12A cells.

(ii) There is a significant correlation (*P* < .05) between the cytotoxic potential of [A], [B], [C] and [D] and their ability to induce the release of cytochrome c in MCF-12A cells.

## 4. DISCUSSION

Results from this study have indicated that [B], [C], and [D] have octanol/water partition coefficients that are lower than that of [A]. These compounds exhibit more selectivity for different cell types but are less intrinsically potent. The lower octanol/water partition coefficients facilitate the more selective uptake of these compounds. On the other hand, Berners-Price et. al. [13] described a related compound, that is, [Au(dpmaaH_2_)_2_]Cl, to be hydrophilic with no significant activity against cancer cells indicating the important role lypophilicity plays in the design of antitumour compounds.

A significant correlation between drug uptake and the octanol/water partition coefficient of compounds has been established [10]. Although [C] and [D] have intermediate partition coefficients we failed to obtain a significant correlation between drug uptake and the octanol/water partition coefficient.

McKeage et al. [4] affirmed that compounds with an intermediate lipophilicity displayed significant antitumour activity, less dose-limiting toxicity, and a higher plasma concentration of gold when compared to more lipophilic or hydrophilic compounds. Similarly, we found a significant correlation between lipophilicity and cytotoxicity. However, results from this study suggest that the cellular uptake of these compounds is not dependent on the lipophilicity of the compound.

The mere fact that cytochrome c is being released indicates that the cell will eventually undergo apoptosis. The uncoupling of oxidative phosphorylation results in the swelling of the mitochondria [5], which consequently causes the outer membrane of the mitochondria to rupture, leading to the release of cytochrome c [7]. In this study, we found a significant correlation between lipophilicity and mitochondrial damage only in the case of the nontumourogenic breast cell line (MCF-12A) and not the breast cancer cell line (MCF-7) indicating a possible selectivity of less lipophilic compounds for mitochondrial cell membranes.

Furthermore, cytotoxicity results indicate that MCF-7 is more sensitive than MCF-12A to [B], [C], and [D].

The results from this study suggest that [C] and [D] act more selectively against a breast cancer and myeloma cell line than [B] but possess less overall cytotoxicity compared to [A], which is an important characteristic in selecting anticancer agents. These compounds possess an intermediate partition coefficient compared to [A] and [B], which plays an important role in their uptake by both normal and cancer cells. Future work will include experimental studies to obtain a clear understanding of the mechanism of action of [C] and [D] and the influence of tin in this regard. Although the high IC_50_ values obtained with [C] and [D] against malignant cell lines are not achievable in vivo, it provides a basis for the synthesis of a new family of compounds with intermediate partition coefficients compared to [A] and [B] but increased activity against cancer cells.

## Figures and Tables

**Table 1:** Average octanol/water partition coefficients of the experimental compounds as well as the mean log octanol/water partition coefficient ± standard error mean (SEM).

Octanol/water partition coefficient and		
mean log octanol/water partition coefficient ± SEM		

Experimental	Average^1^ octanol/water	Mean log octanol/water
compounds	partition coefficient	partition coefficient
	value	± SEM
[A]	12.06	1.07 ± 0.104
[B]	0.038	−1.568 ± 0.149
[C]	2.3	−0.012 ± 0.317
[D]	1.67	0.09 ± 0.155

^1^ Average of 9 experiments at three different concentrations.

**Table 2:** Mean drug concentration (*μ*M) causing 50% cell death (IC_50_) after treatment of various cell lines with the five experimental compounds.

Cell lines	[A]^1^	[B]	[C]	[D]
MCF-7	2.31	20.327	5.33	6.508
MCF-12A	2.424	160	25.639	16.58
A2780	1.158	22.244	12.996	17.277
A2780cis	2.58	37.663	10	20
HeLa	1.407	63.793	11.476	12.264
CoLo	7.686	200	31.141	26.234
Primary fibroblasts	0.887	170	11.874	8.046
B16	0.115	49.89	4.999	2.738

^1^ Average of 3–5 experiments.

**Table 3:** Gold uptake (mg/L) by MCF-7 and MCF-12 A cells treated with the experimental compounds.

Gold Uptake (mg/L)^1^				

Cell lines	[A]	[B]	[C]	[D]
MCF-7	5.27	5.19	2.85	2.68
MCF-12A	6.51	6.83	3.3	2.63

^1^ Average of 3 different experiments.

**Table 4:** Mitochondria of MCF-7 and MCF-12A cells with undamaged membranes after treatment with experimental compounds.

Percentage mitochondria with undamaged membranes					
calculated as the invert of the percentage cytochrome c release^1^					

Cell line	Untreated	[A]	[B]	[C]	[D]
MCF-7	60.75	3	61.75	39.5	41.0
MCF-12A	70.25	2.25	76.5	48.0	46.25

^1^Average of 3–5 experiments.
